# Computational modeling and experimental analysis for the diagnosis of cell survival/death for Akt protein

**DOI:** 10.1186/s43141-020-00026-w

**Published:** 2020-04-21

**Authors:** Ayodeji Olalekan Salau, Shruti Jain

**Affiliations:** 1 Department of Electrical/Electronics and Computer Engineering, Afe Babalola University, Ado-Ekiti, Nigeria; 2 Department of Electrical and Communications Engineering, Jaypee University of Information Technology, Solan, Himachal Pradesh India

**Keywords:** AKT, Apoptosis, Eigenvalues, Protein, Neural network

## Abstract

**Background:**

Signalling systems that control cell decisions allow cells to process input signals by apprehending the information of the cell to give one of these two feasible outputs: cell death or cell survival. In this paper, a well-structured control design methodology supported by a hierarchical design system was developed to examine signalling networks that control cell decisions by considering a combinations of three primary signals (input proteins): the pro survival growth factors, *epidermal growth factor* (EGF), insulin, and the pro death cytokine, *tumour necrosis factor-α* (TNF), for AKT/protein kinase B. The AKT actions were examined by using the three input proteins for cell survival/apoptosis for a period of 0–24 h in 13 different slices for ten different combinations.

**Results:**

Experimental analysis was performed to consider the reactions that were essential to explain the action of AKT. Furthermore, pre-processing and data normalization were performed by using standard deviation, plotting histograms, and scatter plots. Feature extraction and selection were performed using correlation matrix. Radial basis function (RBF) and multiple-layer perceptron (MLP) were used for cell survival/death classification. For all the ten combinations of the three input proteins, 42.85, 347.22, 153.13 were obtained as the minimum value, maximum value, and mean value, respectively, and 126.11 was obtained as the standard deviation for 5-0-5 ng/ml combinations of TNF-EGF-Insulin. The results obtained with MLP 10-8-1 were found to outperform other techniques.

**Conclusion:**

The results from the experimental analysis indicate that it is possible to build self-consistent compendia cell-signalling data based on AKT protein which were simulated computationally to yield important insights for the control of cell survival/death.

## Background

Computational biology has recently emerged with a system-level understanding of biological processes. Biological signalling networks process extracellular cues to control the cell divisions such as growth-quiescence, survival/death, and proliferation-differentiation [1]. There are different profound and exciting issues which can be considered. These include robustness of network structures, biological systems and dynamics, and applications to drug discovery. Bioelectronics is a field of electronics that encompasses a range of biology and electronics topics. One aspect of bioelectronics is processing biological systems in electronic applications (e.g., processing novel electronic components from DNA, nerves, or cells) [2]. It also focuses on physically interfacing electronic devices with biological systems such as cell-electrode, brain-machine, or protein-electrode. Applications in this area include supportive technologies for individuals with brain-related disease or injury, namely: paralysis, artificial retinas, and new technologies for protein structure-function measurements.

In recent years, protein kinase small cell inhibitors have been considered as a new area of interest in the diagnosis of cell survival/death [3, 4]. Many such kinases are used by clinicians for the treatment of cancer, chronic inflammatory disease, etc. Cancer can be characterized as a genetic disease [5]. There are three types of genes that are affected: tumour suppressor genes, oncogenes, and stability genes. The classical tumour suppressor genes “RB1” play a major role in controlling cell cycle. Informally, they are present in the retinoblastoma and other tumours. Apoptosis and necrosis are two different forms of cell death. Necrosis is an early disruption of the cell membrane and is associated with organelle swelling, while apoptosis activates the energy required for intracellular interaction which is tightly regulated and conserved throughout evolution. The progressive series of biochemical and morphological changes on cell surfaces of phosphatidylserine, to proteolytic cleavage of numerous intracellular proteins, to nuclear condensation and fragmentation, and the cleavage of DNA into nucleosomal fragments are known as apoptotic cell death [6–8]. One of the important researches in apoptotic cell death is cancer [9]. All the strategies used for killing the tumour/cancer cells are called anticancer strategies and have been used in clinical oncology, for example, gamma-irradiation, chemotherapy, or immunotherapy. Intrinsic and extrinsic pathways are associated with the stimulation of cell death signal pathways in cancer cells [10].

This paper therefore considered the AKT protein pathways that control cell death/survival decisions using a combination of three input proteins, namely *tumour necrosis factor-α* (*TNF*), *epidermal growth factor* (*EGF*), and *insulin*. There are three isoforms of AKT: AKT1, AKT2, and AKT3. For mouse studies, AKT1 is used for cell survival; AKT2 is primarily used for glucose homeostasis, while AKT3 is mainly used for brain development and has a more preponderant role in triple negative tumours. The over-activation of AKT is generated with a myristoylation sequence leading to myrAKT1 or myrAKT2 which maintain the constitutively active protein at the cell membrane. Down-regulation of AKT is generated with shRNA constructions (shAKT1 or shAKT2). The serine PKB/AKT/Rac are the initial identified oncogene and kinase with similar properties as of PKC/PKA. They also play a major role in coordinating the progression of survival, metabolism, and death using the three input proteins (signalling pathways). PI3K/AKT signalling cascade is activated by three signalling pathways through respective tyrosine kinase-like neurotrophin receptors (TrK’s). After dimerization, PI3K gives phosphoinositide phosphates (PIP2 and PIP3) at the inner side of the plasma membrane. Phosphoinositide-dependent protein kinase 1 (PDK1) later works together with PIP2 and PIP3 to phosphorylate to activate AKT. In order to perform various functions in the cell, AKT was able to phosphorylate a wide variety of substrate protein. Synthesis peptides with a sequence related to the phosphorylation of GSK3 acts as substrate of AKT kinase activity. AKT is the major factor in different types of cancer. AKT signalling network have diverse downstream effects on cellular metabolism, through either direct regulation of nutrient transporters and metabolic enzymes or the control of transcription factors that regulate the expression of key components of metabolic pathways. It regulates cell growth, survival, and metabolism from exogenous growth stimuli. The molecular events controlling cellular metabolism downstream of PI3K and AKT which represent two major hallmarks of cancer are: growth factor independence through oncogenic signalling and metabolic reprogramming to support cell survival and proliferation. AKT activates NFκB by regulating IKB kinase which results in transcription of pro-survival genes.

This paper examines the cell death/survival decisions for AKT protein pathways. Different parameters were calculated for all the ten different combinations of three inputs proteins. Out of which the best combination was selected using correlation matrix and the results were validated by calculating their Eigenvalues. The selected concentrations were classified using artificial neural networks (ANN). A time series 3D plot was generated for all the best combinations and validated with the training and testing accuracies. The training and testing results yield the same results as that of the neural network (NN) model which accurately predicts cell survival or otherwise cell death. The hallmark of this work is in the description of the predictive model of a cytokine-signal-response compendium used to investigate the regulation of cell fate with the combination of the input proteins for AKT protein.

The rest of this paper is organized as follows. In the “Methods” section, the materials and methods employed for modeling and experimental analysis for the diagnosis of cell survival/death for AKT protein were described in detail. The “Results” section explains the results obtained, and thereafter the results were discussed. This is followed by conclusion and recommendation for future work in the “Conclusion” section.

## Methods

**Fig. 1:**
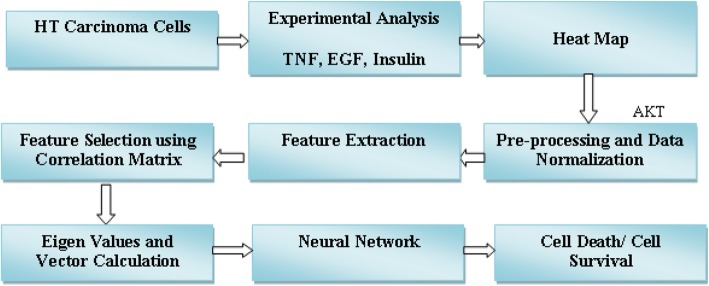
Proposed methodology for cell death/survival decision for AKT protein

In this section, experimental analysis was performed on HT carcinoma cells. A heat map was obtained from the analysis of ten different concentrations of the three input proteins. Later, features were extracted and selected using a correlation vector. The selected features were classified using different neural network techniques, namely: multiple-layer perceptron (MLP) and radial based function (RBF). The block diagram of the proposed methodology is shown in Fig. 1. The prediction model for cell death/survival was implemented with the proposed method using Statistica Software. In total, we obtained 300 values for each combination of input proteins.

**Fig. 2:**
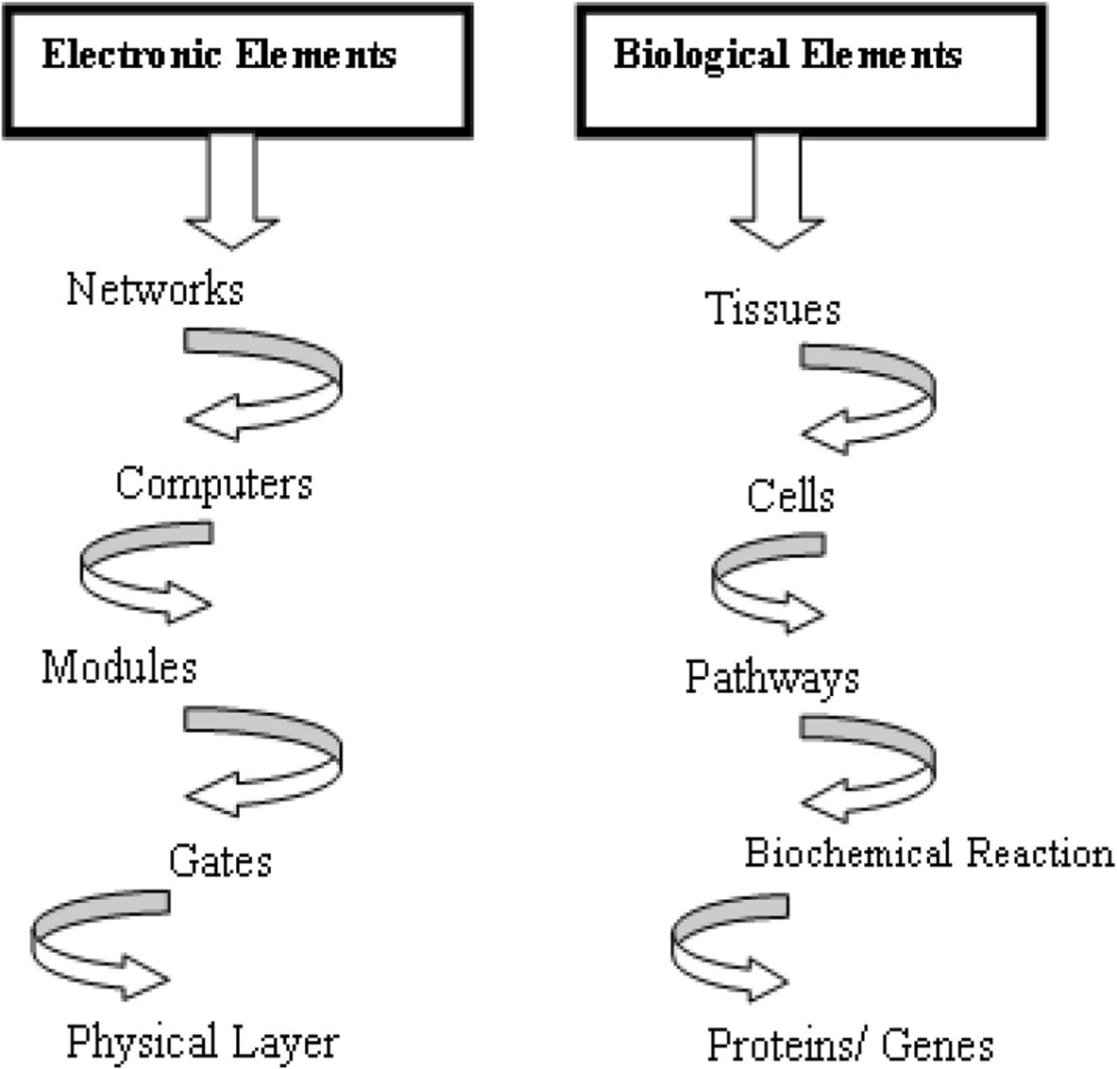
Proposed hierarchical model of electronic and biological elements

Studies of signalling pathways are focused on depicting downstream and upstream interactions, and then systemizing these interactions into linear cascades that balance information from cell surface receptors to cellular effectors. A bottom-up approach was used for the hierarchical model as shown in Fig. 2.

The bottom-up hierarchical approach starts with the proteins/genes as biological components analogous to the physical layer which consists of active and passive components from electronic elements. In the hierarchy, the next layer is the device layer which comprises of biochemical reactions that regulate the flow of information and manipulate physical processes. The biochemical reactions are equivalent to logic gates which perform computations in a computer. At the module layer, a synthetic biologist could use quiet a number of biological devices to assemble complex pathways that function like integrated circuits. The connection of these modules to each other and their integration into host cells allows the synthetic biologist to extend or modify the behaviour of cells in a programmatic fashion.

**Fig. 3:**
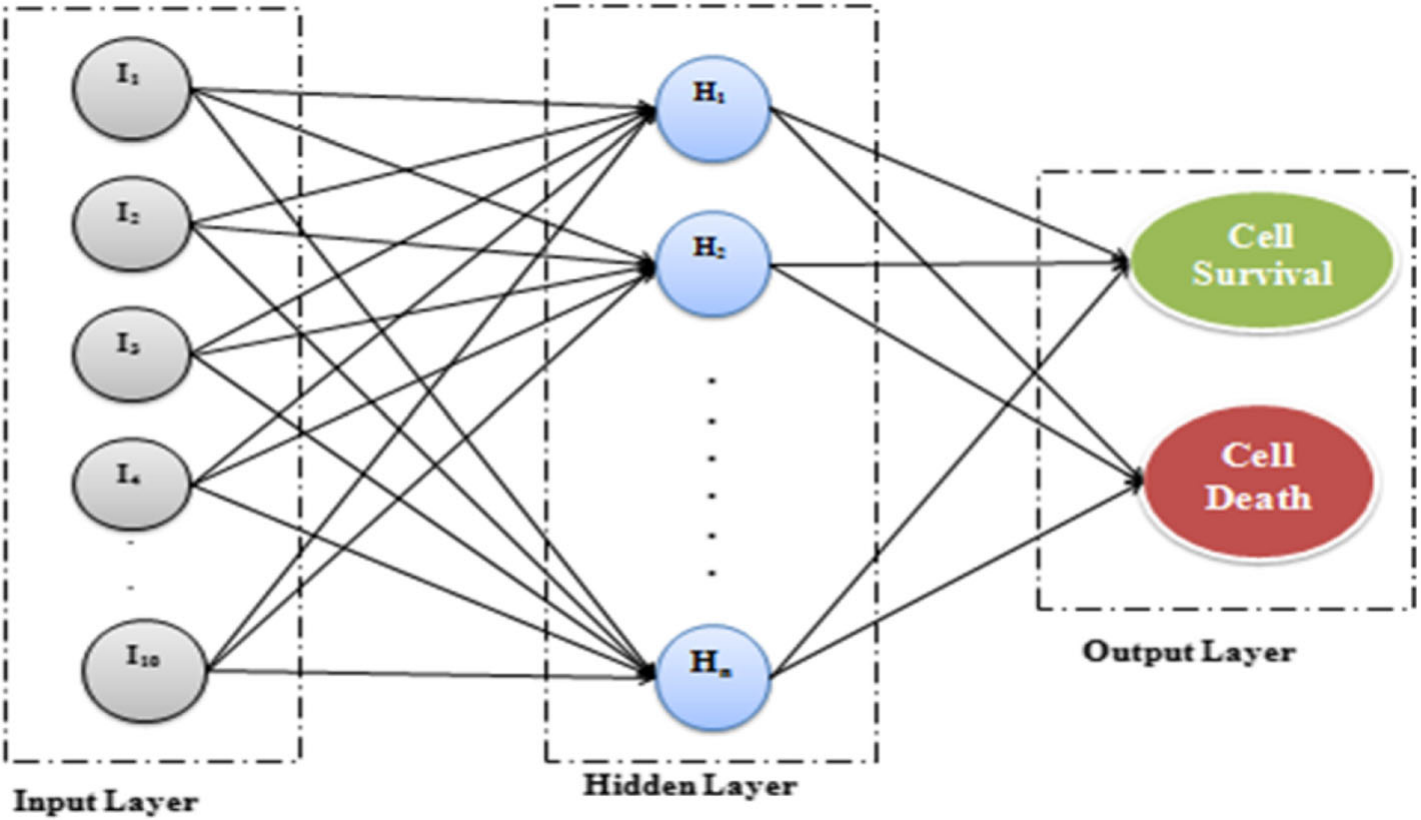
Proposed ANN model for the detection of cell survival/death for AKT

HT carcinoma cells are considered for the analysis of cell survival/death by using AKT as a marker protein. The experimental analysis was performed by considering different concentrations of TNF such as 0, 0.2, 5, and 100 ng/ml. Similarly, different concentrations of EGF, such as 0, 1, and 100 ng/ml, and insulin, such as 0, 1, 5, and 500 ng/ml, for making different cultures were analysed for a period of 24 h by adding 1/20 of diluted stimulus. The 0–24 h time frame was divided into 0, 5, 15, 30, 60, and 90 min, and 2, 4, 8, 12, 16, 20, and 24 h. The cells were exposed to ten cytokine treatments so as to explore systematically the relationship between activation of intracellular signalling cascaded as cytokine receptor interaction and survival death cell fate decisions. All the observations were monitored for a period of 48 h. To explore systematic relationships between the activation of intracellular signalling cascades, cytokine receptor interaction, and apoptosis-survival cell fate decisions cells were exposed to a set of ten different treatments of input proteins. At the 13 time point after cytokine addition, three replicate dishes of cells were harvested to measure kinase activities. Altogether, ten distinct protein signals were examined, namely, (a) assayed in vitro using microtiter-based immuno-complex kinase activity assays: ERK, JNK1, AKT, MK2, and IKK; (b) antibody arrays: phospho-to-total (pt) and phospho total measures of EGFR and AKT; and (c) immunoblotting: five phosphorylation sites on four proteins. Out of the different proteins, AKT signals were examined. Each protein signal was integrated by 12-h, 24-h, and 48-h time frame and then analysed with a set of three input protein treatment. This analysis generated a heat map in which the positions of ten protein signals were defined in comparison to the TNF, EGF, and insulin stimuli. The heat map was prepared for the marker protein of ten different concentrations of input proteins. The ten different concentrations of input proteins (TNF-EGF-insulin) are: 0-0-0, 5-0-0, 100-0-0, 0-100-0, 5-1-0, 100-100-0, 0-0-500, 0.2-0-1, 5-0-5, and 100-0-500. Histograms, standard deviation, and scatter plots were calculated to pre-process the data. Different features like mean, maximum, minimum, and standard deviation for training, testing, validation, and overall data were calculated for all ten different concentrations of the input combinations. Correlation vectors were calculated as feature selection techniques and were used to select the best concentrations of input combinations [11]. The results were validated using Eigenvalues and vector calculations. With the help of Eigenvectors, linear transformation is easy to understand. An eigenvector *ν* of a matrix *A* is independent of the linear transformation: Aν = λν ⇒ λ(B*u*) = A(B*u*). Eigenvectors are a set of basic functions that help in describing data variability. The Eigenvalues of our data were calculated from the best combinations of three input proteins which were used to classify cell death/survival for AKT protein. For classification of the proteins, we have employed artificial neural network (ANN) techniques such as MLP and RBF for cell death and cell survival decisions. ANN is a special nonlinear model for classification, clustering as well as regression. There are at least three layers of nodes for a MLP, namely: input layer, hidden layer, and output layer. The *input layer* consist of input variables which are numeric. Non-numeric data is converted to numeric before it can be used in an ANN technique. This layer is sometimes called the visible layer. The *hidden layers* consist of layers of nodes between the input and output layers; there may be one or more of these layers. The *output layer* is a layer of nodes which produce the output variable. Our proposed ANN model for the detection of cell survival/death for AKT is shown in Fig. 3.

ANN techniques are fast becoming a useful approach for signal-processing technologies. In engineering, neural networks serve two important functions: as nonlinear adaptive filters and as pattern classifiers. They are most often adaptive nonlinear systems that learn to perform a function (an input/output map) from data. Adaptive implies that the system parameters change during operation, normally called the training phase. After the training phase, the ANN parameters are fixed and can be deployed to solve problems.

## Results

**Fig. 4:**
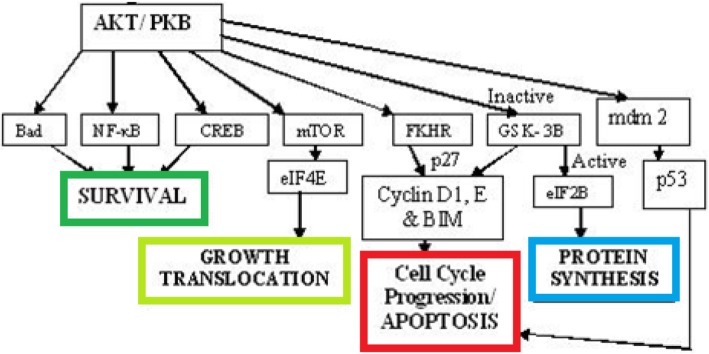
Pathway for cell survival/death for AKT

The experimental observation of cell death/survival from cells treated with ten cytokine combinations of TNF, EGF, and insulin by using AKT was presented in this section. AKT proteins form signalling networks which lead to cell survival/death as shown in Fig. 4 [12].

Futhermore, a similar experimental analysis was carried out as performed in [13, 14]. The results obtained show high similarity. The experimental analysis shows that it is possible to build self-consistent compendia cell-signalling data based on AKT protein which were simulated computationally to yield important insights into the control of cell survival/death. For the purpose of analysis, different experiments were performed with ten different concentrations of three input proteins for 0–24 h in 13 different slices of AKT protein. The novelty of this work lies in the threefold marker protein selection technique; the first stage includes pre-processing techniques, followed by extraction of different features like minimum, maximum, mean, and standard deviation values to select the best combinations of TNF-EGF-Insulin, and lastly, detection was performed using ANN in the third stage to provide a high detection accuracy and low complexity. The proposed method when tested on AKT protein shows that the MLP provides better results with the least run-time complexity for cell survival/death detection. Since ANN techniques are adaptive to complex problems, by changing the networks topology, they are able to handle different levels of complexity and predict the desired output of a system when adequate experimental data is provided. One of the advantages of ANNs is it allows the modeling of physical phenomena in complex systems without requiring exhaustive experiments or without requiring explicit mathematical representations.

A nonlinear ANN was employed in this study to uncover important aspects of biological cue-signal-response systems using TNF-, EGF-, and insulin-mediated response of HT-29 human colon carcinoma cells. Although several analyses were performed, the hallmark of this work is in the description of the predictive model of a cytokine-signal-response compendium used to investigate the regulation of cell fate with the combination of the input proteins for AKT protein. The compendium contains more than 10,000 biochemical measurements based on the states and activities of cell-signalling proteins and apoptotic responses in human cells. Experimental databases are common in genomics, majorly because sequence data are structured and homogeneous, with clear start and finish points, and the ease to fuse data. In contrast, cell-signalling data are unstructured and heterogeneous and depend on biological content.

## Discussion

**Fig. 5:**
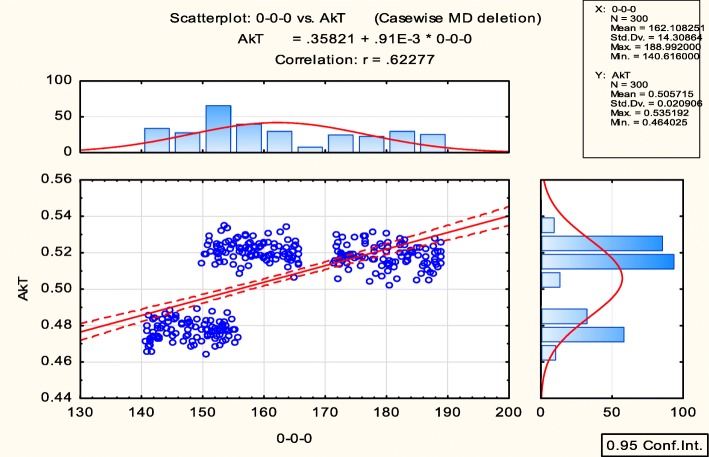
Pre-processing of AKT

After analysis, four output cellular responses (phosphatidylserine exposure (PE), membrane permeability (MP), nuclear fragmentation (NF), and caspase substrate cleavage (CC)) were obtained and used to predict cell death/survival with the consideration of three input proteins (TNF, EGF, and insulin) using a system biology approach (hierarchical model). Ten different concentrations of three inputs and an average of four outputs were analysed and then normalized giving a final result of 10 inputs and 1 output. Furthermore, a heat map in form of an image was prepared and pre-processed by plotting histograms and scatter plots as shown in Fig. 5. The features like mean, maximum, minimum, and standard deviation for training, testing, validation, and overall data were calculated for the ten different concentrations which are presented in Table 1.

**Table 1:** Various extracted features for AKT proteins

Samples	Data statistics (AKT data)										
	0-0-0	5-0-0	100-0-0	0-100-0	5-1-0	100-100-0	0-0-500	0.2-0-1	5-0-5	100-0-500	AKT
Minimum (train)	140.62	164.91	83.77	161.62	81.72	151.16	140.69	143.92	42.85	110.21	0.47
Maximum (train)	188.99	188.99	155.71	205.55	156.59	178.01	217.53	200.24	346.22	323.42	0.54
Mean (train)	161.66	177.72	108.54	182.22	122.48	165.65	176.99	173.12	160.28	221.72	0.51
Standard deviation (train)	13.77	6.03	26.16	12.19	28.25	6.73	24.66	18.02	128.77	80.89	0.02
Minimum (test)	140.79	166.35	83.79	162.51	82.19	151.19	141.25	143.80	42.98	110.72	0.47
Maximum (test)	188.78	188.91	154.21	205.73	154.65	178.10	215.79	198.38	347.22	323.40	0.53
Mean (test)	162.04	176.50	108.68	182.80	119.59	166.54	177.30	170.44	171.75	230.53	0.51
Standard deviation (test)	14.74	6.22	27.11	11.85	28.08	7.24	24.91	18.61	132.39	80.82	0.02
Minimum (validation)	141.43	165.88	84.12	161.86	83.01	151.83	142.93	143.87	42.98	110.81	0.46
Maximum (validation)	188.35	188.74	155.17	203.85	153.44	176.94	211.48	199.64	336.94	315.43	0.53
Mean (validation)	164.25	179.94	119.00	179.56	134.49	164.93	166.15	180.60	101.12	195.40	0.50
Standard deviation (validation)	18.16	4.80	28.95	5.78	4.96	6.67	18.30	5.65	16.63	38.47	0.02
Minimum (overall)	140.62	164.91	83.77	161.62	81.72	151.16	140.69	143.80	42.85	110.21	0.46
Maximum (overall)	188.99	188.99	155.71	205.73	156.59	178.10	217.53	200.24	347.22	323.42	0.54
Mean (overall)	162.11	177.87	110.13	181.91	123.84	165.68	175.41	173.84	153.13	219.09	0.51
Standard deviation (overall)	14.31	6.15	26.88	11.84	27.40	6.74	24.24	17.86	126.11	79.95	0.02

Table 1 shows the mean training, mean testing, mean validation, mean overall data, maximum training, maximum testing, maximum validation, maximum overall data, minimum training, minimum testing, minimum validation, minimum overall data, standard deviation training, standard deviation testing, standard deviation validation, and standard deviation overall data for all the ten different combinations of three input proteins for AKT protein. For all the ten concentrations of the three input proteins, 42.85, 347.22, 153.13 were obtained as the minimum value, maximum value, and mean value, respectively, and 126.11 as the standard deviation for 5-0-5 ng/ml combinations of TNF-EGF-Insulin. In addition, the best combinations were selected by using the correlation matrix as presented in Table 2. This matrix is used to describe the dependency between different data sets. In cases of analysis dealing with numerous secondary variables, the correlation matrix is used to describe this dependency effectively.

**Table 2:** Correlation matrix for AKT protein

Variable	*P* < 0.050, *N* = 300											
	Means	Std. Dev.	0-0-0	5-0-0	100-0-0	0-100-0	5-1-0	100-100-0	0-0-500	0.2-0-1	5-0-5	100-0-500
0-0-0	162.11	14.31	1.00	0.09	0.85	0.14	0.04	0.57	− 0.53	− 0.02	− 0.07	0.40
5-0-0	177.87	6.15	0.09	1.00	0.31	− 0.49	0.53	-0.16	− 0.47	0.50	− 0.53	− 0.44
100-0-0	110.13	26.88	0.85	0.31	1.00	− 0.23	0.45	0.38	− 0.84	0.37	− 0.48	0.01
0-100-0	181.91	11.84	0.14	− 0.49	− 0.23	1.00	*− 0*.*87*	0.45	0.62	− 0.84	0.87	0.86
5-1-0	123.84	27.40	0.04	0.53	0.45	*− 0*.*87*	1.00	-0.37	*− 0*.*81*	0.95	*− 0*.*99*	− 0.87
100-100-0	165.68	6.74	0.57	− 0.16	0.38	0.45	− 0.37	1.00	− 0.03	− 0.38	0.35	0.61
0-0-500	175.41	24.24	− 0.53	− 0.47	− 0.84	0.62	*− 0*.*81*	-0.03	1.00	− 0.74	0.83	0.48
0.2-0-1	173.84	17.86	− 0.02	0.50	0.37	− 0.84	0.95	-0.38	− 0.74	1.00	*− 0*.*95*	− 0.87
5-0-5	153.13	126.11	− 0.07	− 0.53	− 0.48	0.87	*− 0*.*99*	0.35	0.83	*− 0*.*95*	1.00	0.87
100-0-500	219.09	79.95	0.40	− 0.44	0.01	0.86	− 0.87	0.61	0.48	− 0.87	0.87	1.00

**Fig. 6:**
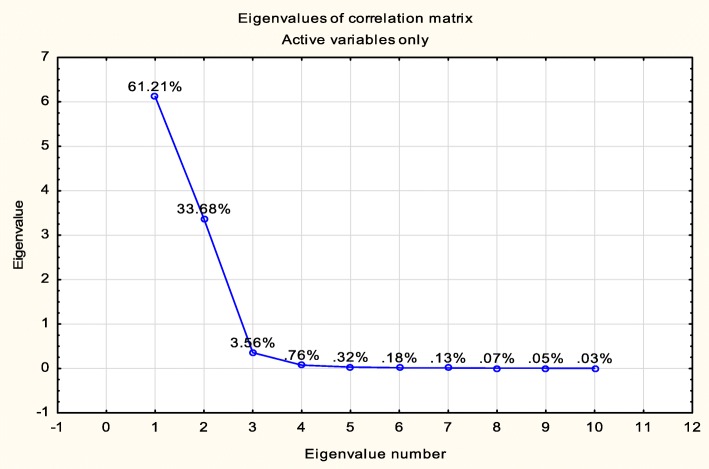
Plot of Eigenvalues for the correlation matrix

From Table 2, it is observed that the 0-0-500 concentration is less correlated with each other. Therefore, this concentration can be neglected for the classification of the cells. To validate the results, Eigenvalues of the correlation matrix were also calculated as shown in Fig. 6.

Eigenvalues are a measure of the data variance which are used to reduce the dimension of large datasets by selecting only a few modes with significant values and are also used to find new variables that are uncorrelated. The selected concentrations were used for the classification of the cells using ANN.

An ANN model was developed for the prediction of cell survival/cell death considering ten different combinations. The authors have implemented the propsed neural network model using STATISTICA 2016 data miner software. The proposed ANN model was developed for the prediction of cell survival/cell death considering ten different concentrations of three input proteins. The NN consists of one input layer with ten nodes, where each node corresponds to the different concentrations. It consists of one hidden layer with different hidden nodes and one output layer with 2 nodes. When the predicted output in the second neural network is > 0.5 it will lead to cell survival; otherwise, it leads to cell death.

**Fig. 7:**
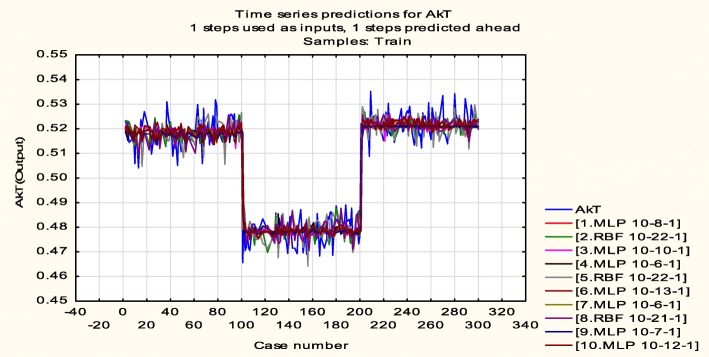
Time series plot using MLP and RBF techniques to obtain the best 10 results. Note: 10 signifies ten different combinations of three different input proteins, 8 hidden layers, and 1 output cell survival/death result

In comparison with the statistical analysis, ANN is a nonlinear model which is easy to use and understand and is mostly used for solving various classification and forecasting problems. The results reveal that the proposed ANN model is most adequate to estimate the physiological functions from intracellular protein expressions. Figure 7 shows the time series plot of the 10 results obtained from the analysis using MLP and RBF techniques. A time series graph or plot is a graphical representation of time series data on the *x*-axis (time increments/cases) and on the *y*-axis, the corresponding measured values are plotted. Time series plots are very useful as they illustrate how the values of the measured variable changes over time. In Fig. 7, the *x*-axis defines the different 300 cases which were considered and the *y*-axis defines the AKT values. Figure 7 further shows the ten different combinations of MLP and RBF. MLP 10-12-1 indicates the input-hidden layer-output.

**Fig. 8:**
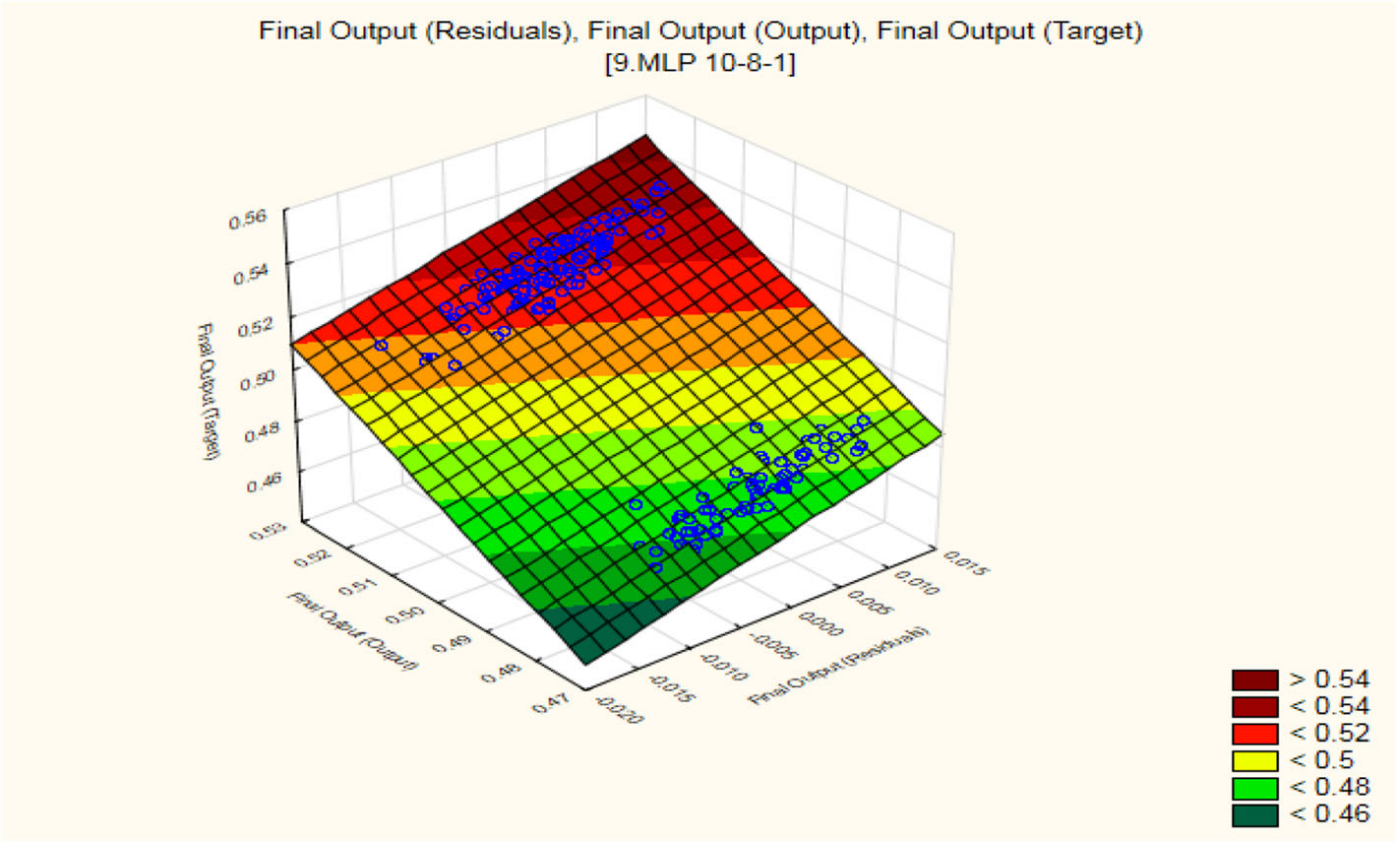
3D plot of MLP 10-8-1 for final output of residual, target, and output

Out of the ten results, MLP 10-8-1 (10 signifies ten different combinations of three different input proteins, 8 hidden layers, and 1 output cell survival/death result) outperforms all other results. The three-dimensional plot for MLP 10-8-1 is shown in Fig. 8 for residual, target, and output. Different threshold values were considered resulting in cases of cell survival/death. Figure 8 also shows the threshold values which are expressed with different colours.

The results were also validated by calculating the training and testing perfections. Table 3 shows the training and testing accuracy of ten different neural networks using MLP and RBF. The table shows that MLP 10-8-1 gives 99.89% for both the training and test accuracies

**Table 3:** Training and testing accuracies using different ANN models for AKT

S/no.	Network name	Training % accuracy	Test % accuracy
1	MLP 10-7-1	99.14	98.94
2	RBF 10-22-1	99.76	99.67
3	MLP 10-10-1	99.77	99.76
4	MLP 10-6-1	99.20	99.22
5	RBF 10-22-1	99.58	99.30
6	MLP 10-13-1	98.48	98.43
7	MLP 10-6-1	94.47	90.58
8	RBF 10-21-1	99.70	99.67
**9**	**MLP 10-8-1**	**99.89**	**99.89**
10	MLP 10-12-1	98.33	98.12

Results obtained in Table 3 validate the values obtained after generating the time series plots as shown in Fig. 7. In addition, the training and testing accuracies yields the same results with the neural network model which accurately predicts cell survival or otherwise cell death. In Table 4, we present a comparison of the results of the proposed method with existing works. The results show that the proposed method outperforms the existing methods for the analysis and determination of cell survival/death.

**Table 4:** Comparison of results of research papers with the proposed technique

S. No.	Technique	Proteins	Accuracy (%)
1.	GLCM + k-NN [15]	EGFR, IRS, ERK, MK2, JNK, FKHR	75.60
2.	GLDS + k-NN [6]	AKT	76.90
3.	DWT (Bior 4.4) + SSVM [7]	ERK, MK2, JNK	80.00
4.	GLDS + SVM [6]	AKT	84.60
5.	GLCM + SVM [15]	EGFR, IRS, ERK, MK2, JNK, FKHR	85.80
6.	**Proposed technique (MLP ANN)**	AKT	99.89

## Conclusion

Biological systems can create complex structures from very simple systems. In this paper, a series of experimental analysis were performed with ten different concentrations of three input proteins for a period of 0–24 h in 13 different slices. Based on the experimental analysis, a heat map (in the form of an image) was generated for different marker proteins. Initially, the data was pre-processed, and subsequently different features were extracted and selected based on the correlation matrix method. The selected features were then validated by calculating their Eigenvalues. Furthermore, RBF and MLP techniques were applied for the cell death/cell survival decisions. A time series 3D plot was generated for all the best combinations and validated with its testing accuracy. In the future, different optimization techniques will be applied for the selection of features.

## Data Availability

All data generated or analysed during this study are included in this published article.

## Abbreviations

ANN: Artificial neural networks
CC: Caspase substrate cleavage
DNA: Deoxyribo nucleic acid
EGF: Epidermal growth factor
ERK: Extracellular signal regulated protein kinase
GSK3: Glycogen synthase kinase 3
JNK1: c-Jun NH2 terminal kinase 1
MK2: Mitogen-activated protein kinase-activated protein kinase 2
MLP: Multiple-layer perceptron
MP: Membrane permeability
NF: Nuclear fragmentation
NFκB: Nuclear factor kappa B
NN: Neural network
PE: Phosphatidylserine exposure
PDK1: Protein kinase 1
PIP: Phosphoinositide phosphates—plasma membrane intrinsic protein. The PIP subfamily is subdivided into two groups: PIP1 and PIP2
PKA: Protein kinase A
PKB: Protein kinase B/AKT protein kinase
PKC: Protein kinase C
Rac: Ras-related C3 botulinum toxin substrate
RBF: Radial based function
TNF: Tumour necrosis factor
